# Persistent response to vemurafenib in metastatic ameloblastoma with BRAF mutation: a case report

**DOI:** 10.1186/s13256-019-2140-6

**Published:** 2019-07-25

**Authors:** Morgane Broudic-Guibert, Jean-Yves Blay, Léa Vazquez, Alexandre Evrard, Marie Karanian, Sophie Taïeb, Natalie Hoog-Labouret, Céline Mahier Ait Oukhatar, Rania Boustany-Grenier, Antoine Arnaud

**Affiliations:** 1 Sainte-Catherine Institut, 250 Chemin de Baigne-Pieds, 84000 Avignon, France; 2 Léon Bérard Center, University Claude Bernard Lyon 1, Lyon, France; 3 University Hospital Center Carémeau, Nîmes, France; 4 Oscar Lambret Center, Lille, France; 5 INCA, Boulogne Billancourt, France; 6 UNICANCER, Paris, France

**Keywords:** Vemurafenib, BRAF, Ameloblastoma, Metastatic, Case report

## Abstract

**Background:**

Ameloblastomas are uncommon locally aggressive tumors of odontogenic epithelium that rarely metastasize. Currently, there is no standard of care for the metastatic forms. Several studies have shown that ameloblastomas frequently have a BRAF mutation.

**Case presentation:**

We report a case of a 33-year-old Caucasian woman with ameloblastoma diagnosed 30 years ago who developed lung metastasis 19 years ago. Systemic oral treatment with vemurafenib, a BRAF inhibitor, was initiated 28 months ago within the AcSé French basket clinical trial of vemurafenib.

**Conclusions:**

The patient has shown a durable clinical, functional, and radiographic partial response with vemurafenib. These observations suggest the possibility of introducing neoadjuvant and/or adjuvant targeted therapy in locally advanced ameloblastoma to improve outcome. BRAF inhibition has proved to be an efficient strategy in patients with a BRAF-mutated ameloblastoma.

## Background

Ameloblastomas are rare odontogenic tumors of epithelial origin that are most frequently located in the posterior mandible. These tumors are locally aggressive and rarely metastasize [1]. Local recurrence of ameloblastic tumors occurs in approximately 20% of patients. These tumors are malignant in less than 2% of cases [1]. Metastasis frequently occurs in the lungs (≥75%) and usually is diagnosed many years after the primary tumor [2]. Surgical resection of the primary tumor with intent to cure is the mainstay of treatment for ameloblastomas and is considered the best approach to prevent recurrence and metastasis [3].

## Case presentation

We present a case of a 33-year-old Caucasian woman diagnosed 31 years ago with a plexiform-type ameloblastoma of the left mandible and treated by surgical resection (R0). Relapse was diagnosed 11 years after surgery. The patient had multiple (> 30) bilateral lung metastases. The diagnosis of ameloblastoma lung metastasis was confirmed by thoracotomy biopsy. Clinically, the patient was asymptomatic with a World Health Organization score of 0. Partial response (PR) to standard systemic chemotherapy has been reported previously [4]. However, several patients also have an indolent evolution of their disease and do not benefit from any specific active treatment. Given the absence of clinical symptoms and the patient’s reluctance to receive treatment with possible adverse effects, systemic chemotherapy was not prescribed. However, close surveillance was initiated. The iterative thoracic computed tomographic (CT) scans showed a very gradual increase in size of some of the pulmonary lesions, but with no new lesion identified.

Fourteen years after her diagnosis of metastasis, the patient experienced dyspnea. Functional respiratory exploration (FRE) identified a restrictive syndrome (forced vital capacity at 74% of the theoretical value) and an obstructive syndrome (with 63% expired flows of the theoretical value).

Rebiopsy of the upper lung lobe by thoracoscopy confirmed the pulmonary metastasis of the ameloblastoma, without ameloblastic carcinomatous transformation. Because the patient remained clinically stable, medical follow-up was continued.

Three years ago, the patient’s effort dyspnea worsened, and thoracic CT scans showed a slow and homogeneous tumor progression with increases in the number and size of the bilateral pulmonary nodules. FREs showed an increased ventilatory restriction (forced vital capacity at 62% of the theoretical value) and bronchial obstruction (expired flows at 48% of the theoretical value).

The patient’s tumor sample was found to be BRAF V600E mutated without other mutation (*KRAS, EGFR* [epidermal growth factor receptor], *ALK, c-Kit*). The patient was included in the AcSé clinical trial on 6 December 2016 after her informed consent was obtained. Treatment with vemurafenib was started.

Initially, vemurafenib was administered at a dose of 960 mg twice daily. During the initial 12 months of treatment, following the occurrence of grade 1–2 arthralgia, nausea, and rash that which led to a transient interruption, the dose was reduced to 720 mg twice daily and finally to 480 mg twice daily with acceptable tolerance.

**Fig. 1:**
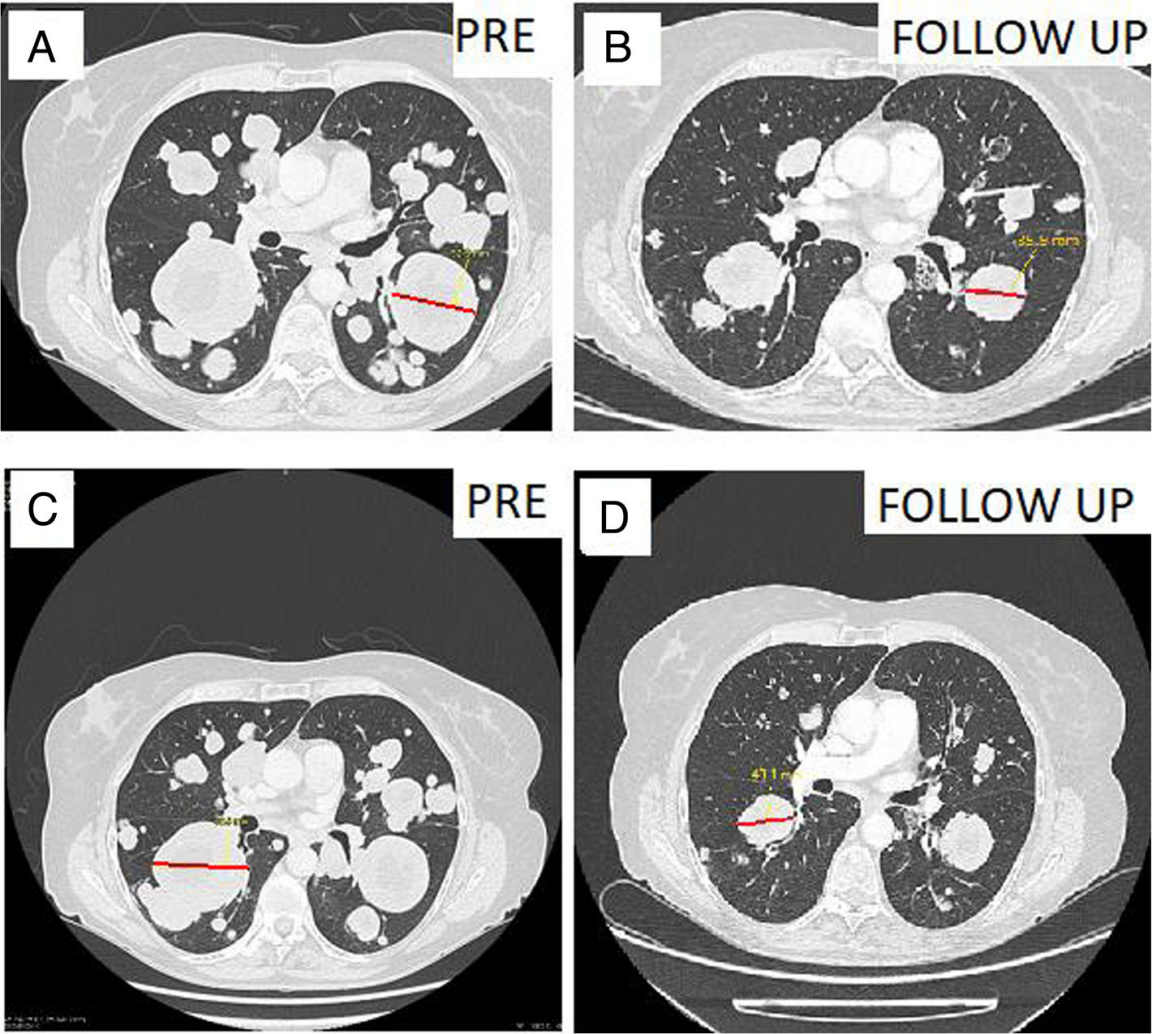
Computed tomographic scan showing response with BRAF inhibitor in a patient with stage IV ameloblastoma. Target lesions were measured before treatment (6 December 2016) and 26 months after treatment (7 February 2018), respectively, at 56 mm (**a**) and 36 mm (**b**) in the left apical lower lobe and 64 mm (**c**) and 43 mm (**d**) in the right apical lower lobe. Reduction in tumor mass is shown. The red lines denotes the length of lesions taken into account respectively 56 mm for **a**, 36 mm for **b**, 64 mm for **c** and 43 mm for **d**

A CT scan after 3.5 months of treatment showed a PR according to Response Criteria in Solid Tumors (RECIST) version 1.1 and after proofreading by an expert radiologist, with a 30% decrease in the sum of the diameter of the lung target lesions compared with the reference scanner. The response was persistent and was still present at the patient’s last evaluation after 26 months of follow-up (Fig. 1). In addition, the patient has reported a marked improvement in respiratory function with a decreased dyspnea and normal FREs (Table 1).

**Table 1:**
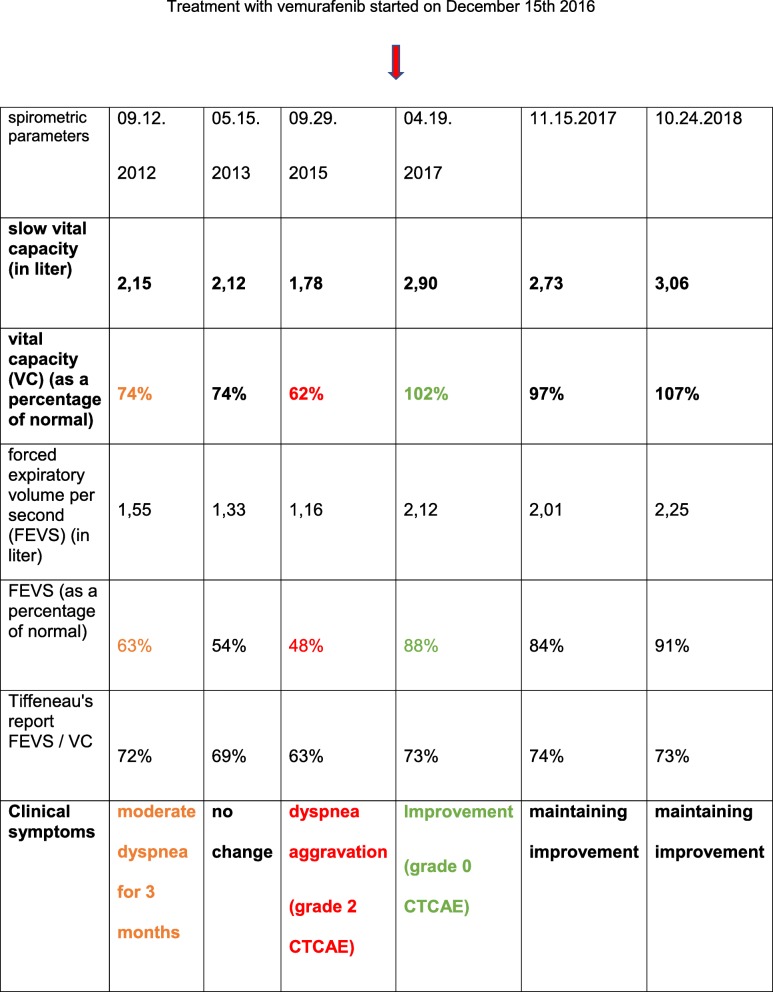
Evolution of functional respiratory explorations before and after treatment with vemurafenib

VC = Vital Capacity, FEVS = Forced Expiratory Volume per second, CTCAE = Common Terminology Criteria for Adverse Events

## Discussion

Ameloblastomas are rare tumors, with a metastatic evolution even rarer. Currently, there is no standard of care for the metastatic forms.

Several studies have shown that BRAF mutation is frequently present in ameloblastomas. In addition, the BRAF V600E mutation is reported to occur in 62% of ameloblastomas and ameloblastic fibromas/fibrodentinomas but is not present in other odontogenic tumors. The BRAF V600E mutation of ameloblastomas was associated with a younger age of onset, with the tumor located in the mandible, and later recurrences, whereas BRAF wild-type tumors arose more frequently in the maxilla and showed earlier recurrences [5, 6]*.*

Targeted agents other than vemurafenib have been explored for treating ameloblastomas. Indeed, Kurppa *et al.* discovered significant EGFR overexpression in ameloblastoma, but the response to EGFR-targeted drugs was variable [7]. These data offer a rationale for testing BRAF inhibitors as novel therapies for ameloblastoma.

Kaye *et al*. also reported a case of ameloblastoma stage IV (local recurrence, cervical metastatic lymph nodes, and pulmonary nodules) that responded well to oral bitherapy with a BRAF inhibitor (dabrafenib) and a mitogen-activated extracellular signal-regulated kinase inhibitor (trametinib) [8]. The treatment improved the patient’s general condition and quality of life. In addition, the patient’s mandibular pain disappeared, the mandibular tumor size and cervical nodes regressed as seen on various imaging examinations, and the hypermetabolism of the pulmonary nodules on the positron emission tomographic/CT scan diminished [8]. However, the authors did not specify whether the tumor response was persistent.

## Conclusions

Although cases of disseminated stage IV ameloblastoma are exceedingly rare, these observations also suggest the possibility of introducing neoadjuvant and/or adjuvant targeted therapy in locally advanced ameloblastoma undergoing surgery to improve outcome and minimize functional and cosmetic morbidity. BRAF inhibition has proved to be an efficient strategy in patients with a BRAF-mutated ameloblastoma.

## Data Availability

The datasets used and analyzed during the current study are available from the corresponding author on reasonable request.

## Abbreviations

ANSM: French National Agency for the Safety of Medicines and Health Product*s*
CT: Computed tomography
EGFR: Epidermal growth factor receptor
FRE: Functional respiratory exploration
PR: Partial response
RECIST: Response Evaluation Criteria in Solid Tumors
